# Guide for Nonequilibrium Molecular Dynamics Simulations of Organic Solvent Transport in Nanopores: The Case of 2D MXene Membranes

**DOI:** 10.1021/acs.jctc.4c00693

**Published:** 2024-11-04

**Authors:** Aysa Güvensoy-Morkoyun, Tuğba Baysal, Ş. Birgül Tantekin-Ersolmaz, Sadiye Velioğlu

**Affiliations:** † Department of Chemical Engineering, Istanbul Technical University, Maslak, Istanbul 34469, Türkiye; ‡ Institute of Nanotechnology, Gebze Technical University, Gebze, Kocaeli 41400, Türkiye; § Synthetic Fuels & Chemicals Technology Center (SENTEK), Istanbul Technical University, Maslak, Istanbul 34469, Türkiye; ∥ Nanotechnology Research Center (NUAM), Gebze Technical University, Gebze, Kocaeli 41400, Türkiye

## Abstract

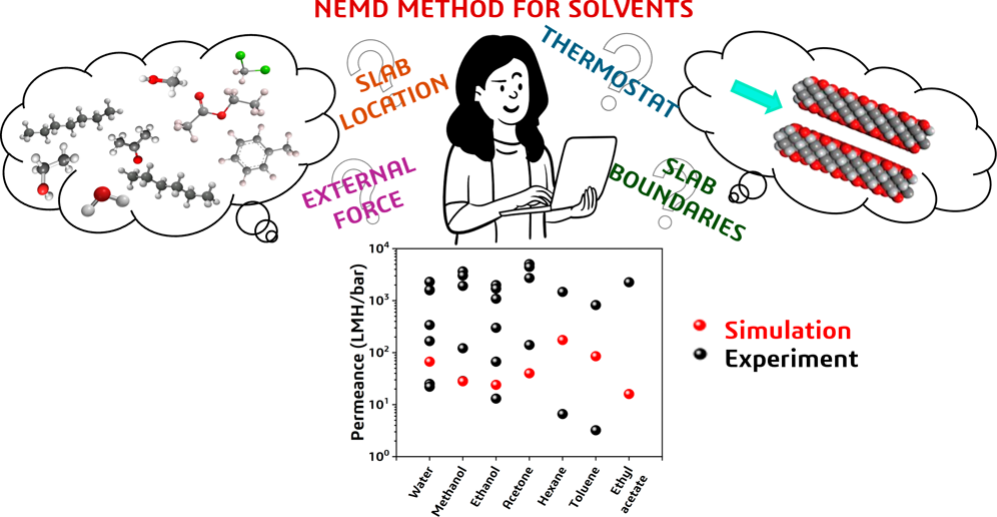

Organic solvent nanofiltration (OSN) stands out as an energy-efficient and low-carbon footprint technology, currently reliant on polymeric membranes. With their exceptional chemical stability and tunable sieving properties, two-dimensional (2D) nanolaminate membranes present distinct advantages over conventional polymer-based membranes, attracting tremendous interest in the OSN community. Computational approaches for designing innovative 2D nanolaminates exhibit significant potential for the future of OSN technology. Imitating the pressure gradient in filtration processes by applying an external force to atoms within a predefined slab, boundary-driven nonequilibrium molecular dynamics ((BD)-NEMD) is a state-of-the-art simulation method with a proven track record in investigating the water transport in nanopores. Nevertheless, implementation of (BD)-NEMD for a broad range of solvents poses a challenge in estimating the OSN performance of theoretical membranes. In this work, we developed a (BD)-NEMD protocol that elucidates the effects of several computational details often overlooked in water simulations but are crucial for bulky solvent systems. We employed a MXene (Ti_3_C_2_O_2_) nanochannel as a model membrane and examined the transport of nine solvents (methanol, ethanol, acetone, *n*-hexane, *n*-heptane, toluene, ethyl acetate, dichloromethane, and water) having different properties. First, the impact of ensemble type, thermostatting, channel wall model, and restraining force constant was elaborated. After optimizing the thermostatting approach, we demonstrated that the location of the force slab particularly affects the flux of bulky solvents by changing the density distribution in the feed and permeate sides. Similarly, the uniformity of intramolecular force distribution in bulky solvents and resulting flux are shown to be prone to manipulation by slab boundaries. Next, the magnitudes of the external force generating a linear relation between the pressure gradient and solvent flux were identified for each solvent to ensure that calculated fluxes could be extrapolated to experimentally related pressures. This linear relation was also validated for a mixture system containing 50% ethanol and 50% water. We then correlated the calculated solvent permeances with various solvent properties, such as viscosity, Hansen solubility parameters, kinetic diameter, and interaction energy. Remarkably, we observed a linear correlation with an *R*^2^ value of 0.96 between permeance and the combined parameter of viscosity and interaction energy. Finally, the solvent permeances calculated with our proposed methodology closely align with the experimentally reported data. Overall, our work aims to serve as a practical guide and bridge the gap in established simulation methods that are suited for a broad range of solvents and membrane materials.

## 1 Introduction

With the USA market size reaching ∼77 billion USD in 2019, a substantial amount of organic solvents is used in the diverse chemical processes employed in the pharmaceutical, petrochemical, fine chemical, dye, and food industries.^1^ Downstream purification and recovery of organic solvents are often considered essential for the economy and environment since the annual solvent release of these industries reaches ∼73 million tons, as reported by the Environmental Protection Agency. Alternative to the current energy-intensive organic solvent separation processes, membrane-based technologies stand out with their simplicity, low energy consumption, cost-effectiveness, and environmental sustainability. Organic solvent nanofiltration (OSN) is a membrane-based, pressure-driven technology that allows the purification of organic solvents and has recently established its role in process intensification and green engineering.^2,3^ Hitherto, a myriad of membrane materials have been fabricated, particularly concentrating on polymer-based materials. However, since polymer-based OSN membranes failed to satisfy the practical requirements, exploring new materials and fabrication strategies for compact and highly ordered microporous membranes has become a persistent pursuit. Among the diverse membrane materials, two-dimensional (2D) nanomaterials respond to this demand owing to their excellent chemical stability under harsh conditions and tunable interlayer spacing, which allows precise molecular sieving. Therefore, studies on 2D OSN membranes have been accelerated recently.^4,5^ While computational design, tailoring, and preliminary testing of novel 2D membranes greatly advance the OSN technology, there is an urgent need for well-established simulation methods to predict the performance of theoretical membranes.

The extensive applications of molecular simulation approaches have paved the way for the characterization of the kinetics, dynamics, and thermodynamics of nanostructures at the molecular scale.^6^ They enable one to mimic the behavior of atomic structures of materials in order to predict their performance and successively fine-tune their structures for specific applications. The success of molecular simulation was also revealed with a proven track record in elucidating the mechanism of fluid transport across several nanopores.^6^ Although the transport across the membranes is driven by pressure or/and concentration difference in filtration experiments, the molecular dynamics (MD) approach, which represents the equilibrium state of the system, was utilized to examine the behavior of water molecules in the nanoconfined region and calculate the equilibrium permeation for biological,^7^ 1D carbon nanotube,^8^ 3D zeolite,^9^ and simplified model pores.^10^ In equilibrium MD simulations, the diffusivity of water is determined from its self-diffusion coefficient without creating any gradient for water transport. Diffusivity is calculated from the mean square displacement of the water molecules captured by the trajectories of a MD simulation. Therefore, in equilibrium MD simulations, only a very slow net water flow through the nanochannel can be observed as a result of thermal fluctuations. Computed water flux cannot be directly used to determine the ability of a membrane to conduct water under a hydrostatic or osmotic pressure difference. To eliminate these shortcomings, a nonequilibrium molecular dynamics (NEMD) methodology was developed where an external force was applied to the feed molecules in order to create a pressure gradient between the feed and permeate streams and membrane material was kept fixed at its initial position to avoid translation of the system along the direction of the external forces. Therefore, the NEMD simulation can simply mimic the actual membrane separation processes. Recently, this approach has been applied intensively to examine the transport of water through several nanomaterials.^11−14^ A few studies also were reported on methanol, ethanol, acetone, acetonitrile, and *n*-hexane transport.^15−18^

Along with solvent permeance, solute rejection is undoubtedly the second most important performance indicator for an OSN membrane. While reliably predicting solute rejection through NEMD simulations proves challenging due to the low concentration of solute molecules and limited simulation time, sampling techniques enable the calculation of the energy barrier of solute transport and elucidate solute exclusion mechanisms in nanopores.^14,19,20^ Moreover, a recent study demonstrated a direct relation between the solvent flux and solute rejection experimentally. Ignacz et al.^21^ proposed the strong impact of average solvent permeance on the average solute rejection based on a large experimental data set consisting of 407 solutes in 11 common solvents for a polyimide-based OSN membrane. Therefore, this study suggests that for a highly permeable OSN membrane, only the measurement of pure solvent permeance can provide insights into the solute rejection performance. That being said, NEMD simulations can greatly expand this type of data set for different materials and solvents once the predicted solvent permeance is correlated with experimentally measured permeance.

In recent NEMD simulations, two approaches, namely, the movable walls (MW)-NEMD and the external field (EF)-NEMD, have been commonly used. (MW)-NEMD approach,^22^ in which molecules are pushed with an external force applied to an impermeable wall in order to accelerate the fluid transport through a membrane, has some disadvantages in the prediction of solvent permeance. Since the simulation time is directly proportional to the fluid bath (volume) on the feed side, it is not possible to run a continuous simulation for a long time or to use the same simulation time for different solvents.^18^ After all of the molecules pass through the membrane material, the simulation terminates. Since the volume of the feed side will drastically change during the short simulation time, the proposed solvent permeance calculated from the initial stage of a (MW)-NEMD simulation does not represent the actual performance of an OSN membrane measured in continuous filtration experiments. Considering the limitations mentioned above in the (MW)-NEMD method, the EF-NEMD approach, which directly exerts a force on all fluid particles and generates a steady-state nonequilibrium flux, was proposed.^23,24^ Shortly after, this method was followed by its modified version, boundary-driven nonequilibrium molecular dynamics ((BD)-NEMD) method, in which a constant force is applied to the fluid in a predefined region of the feed bath.^13,25,26^ Since the permeated solvent molecules return to the feed bath due to the periodicity, a sufficiently long simulation time can be performed by (BD)-NEMD, contrary to the (MW)-NEMD approach. Even so, this approach was mainly applied to examine the water transport,^12,14,19^ except for a single report on acetone.^17^ According to our knowledge, no efforts have been made to predict the permeance of bulky solvent molecules. The main reason for this shortcoming is the lack of a well-accepted methodology that suits a broad range of solvents in the application of this newly developed NEMD concept. In this study, we aimed to develop a methodology and present a guide that can be safely used for different solvents and membrane materials. As a proof of concept, the MXene (Ti_3_C_2_O_2_) nanochannel was considered as a model 2D OSN membrane, and transport of 9 solvents (methanol, ethanol, acetone, *n*-hexane, *n*-heptane, toluene, ethyl acetate, dichloromethane, and water) having different properties was investigated. To propose a feasible methodology, we outlined the encountered problems and provided a step-by-step optimization of the method in a detailed and comparative manner.

## 2 Computational Details

In order to calculate solvent permeance, the recently developed (BD)-NEMD method is employed. In this method, a high-pressure difference is applied to obtain statistically meaningful data within a reasonable simulation time. A pressure difference is introduced by applying an external force to solvent molecules within a predefined slab and permeating molecules are counted.^17^ As a model membrane structure, Ti_3_C_2_T_*x*_ from the MXene family, one of the commonly studied members, is selected since it is a particularly attractive membrane nanomaterial with its high hydrophilicity, easy processability, and tunable interlayer properties. It has been proposed from XPS analysis that surface terminations denoted as T_*x*_ are a combination of =O, −OH, and −F groups with varying stabilities.^27^ In aqueous medium, −F terminal groups are reported to be readily converted into −OH groups. Similarly, −OH groups were found to be transformed into =O at elevated temperatures.^28^ Therefore, the terminal group represented by T_*x*_ in the Ti_3_C_2_T_*x*_ nanolaminate is modeled as =O in our simulations. Pure solvent permeances of 9 solvents (namely, water, methanol, ethanol, acetone, dichloromethane, ethyl acetate, toluene, *n*-hexane, and *n*-heptane) are calculated for the Ti_3_C_2_O_2_ structure.

### 2.1 Modeling the Solvents and Ti_3_C_2_O_2_ MXene

The reliability of the molecular simulations is greatly affected by the modeling of the studied molecules. Charge calculations and force field parameters are important aspects since they govern the interactions between solvent molecules and nanochannels. Therefore, before the system design for simulations, we simulated pure solvents and compared their calculated densities with the experimental densities to verify our solvent modeling approach. For the modeling of water molecules, the widely used extended simple point charge model (SPC/E) was used.^29^ For other solvents, first, the optimized geometry was obtained from "Automated Force Field Topology Builder and Repository".^30^ Then, OPLS-AA force field parameters and charges were obtained from the LigParGen web server.^31−33^ Initially developed for the parametrization of small organic molecules, the OPLS-AA force field also focused on accurately reproducing the experimental heat of vaporization and density of the solvents. For the calculation of partial charges, the OPLS-AA force field was tested for CM*x* models (e.g., CM1A and CM5) which are quantum mechanical-based point-charge methods.^33^ LigParGen web server currently offers two charge models: 1.14*CM1A and 1.14*CM1A-LBCC, while the latter is the modified version of the former via localized bond charge corrections. For many of our solvents (e.g., dichloromethane, acetone, ethyl acetate, methanol, ethanol, and toluene) densities obtained via both charge models were reported;^32^ therefore, we selected the charge model, which reproduces the experimental density with a minimum error.

After charges and force field parameters were obtained, solvent molecules were packed in a cubic simulation box having dimensions of 25 × 25 × 25 Å^3^ using PACKMOL software.^34^ Since the molecular size of each solvent is different, the number of molecules packed in the constant volume varies (Table 1). MD simulations were carried out via a Large-scale Atomic/Molecular Massively Parallel Simulator (LAMMPS).^35^ Lennard-Jones (LJ) 12-6 potential and particle–particle particle–mesh (PPPM) summation were used to model van der Waals interactions and electrostatic interactions, respectively, with a 12.0 Å cutoff. Temperature and pressure were controlled via the Nosé–Hoover algorithm, and a time step of 1 fs was used. Following the minimization, the system was heated from 30 to 298 K in a constant-pressure, constant-temperature ensemble (*NPT*) at 1 bar for 100 ps. Then, 10 ns *NPT* simulation was performed at 298 K. Equilibrated density was reported and compared with the experimental density in Table 1. Dodda et al.^32^ tested OPLS-AA 1.14*CM1A and 1.14*CM1A-LBCC models for 153 solvents and the error range between simulated and experimental densities was found between 0.0 and 14.0% (see Figure S1 in the Supporting Information for error distribution of 153 solvents, illustrated considering the data published in the study of Dodda et al.^32^) which covers our error range as well. Hence, the simulated densities derived from the proposed solvent models closely align with experimental data, confirming the cautious approach taken with these models.

**Table 1:** Densities Obtained via Simulation of Pure Solvent Molecules to Verify the Charge Model and Force Field Parameters

solvent	model	number of molecules^a^	calc. density^b^ (g cm^–3^)	exp. density^b^ (g cm^–3^)	error %
water	SPC/E	417	0.996	0.998	0.200
dichloromethane	OPLS-AA 1.14*CM1A-LBCC	147	1.283	1.394	7.991
acetone	OPLS-AA 1.14*CM1A	127	0.782	0.785	0.382
ethyl acetate	OPLS-AA 1.14*CM1A	96	0.929	0.894	3.915
methanol	OPLS-AA 1.14*CM1A-LBCC	232	0.738	0.787	6.226
ethanol	OPLS-AA 1.14*CM1A	161	0.767	0.785	2.253
toluene	OPLS-AA 1.14*CM1A	89	0.872	0.862	1.147
heptane	OPLS-AA 1.14*CM1A	64	0.674	0.679	0.736
hexane	OPLS-AA 1.14*CM1A	72	0.654	0.654	0.070

a Number of solvent molecules packed within a 25 × 25 × 25 Å^3^ simulation cell.

b Calculated and experimental densities at 298 K.

The crystal structure of Ti_3_C_2_O_2_ was obtained from literature^36^ to generate a primitive cell consisting of seven atoms with lattice parameters *a* = 3.04196, *b* = 3.04189, *c* = 40.02045, α = 90.26414, β = 84.98888, and γ = 120.00138. Partial atomic charge distribution for the primitive cell (see Figure S2 in the Supporting Information) was calculated via the DFT method using the B3LYP functional and DND basis set using the DMOL3 module of the Materials Studio software package (Accelrys Software Inc.). Then, a supercell having two Ti_3_C_2_O_2_ nanolayers with dimensions of 30 × 30 × 34 Å^3^ was constructed by periodically multiplying the primitive cell. The interlayer distance between each Ti_3_C_2_O_2_ nanolayer was set to 7.5 Å (Figure S2) since this is the average interlayer distance for different MXene types reported in the literature.^37−39^ For the testing of 9 different solvents, the interlayer distance was fixed in order to exclude the effect of the channel size and focus on interactions between the MXene and solvent molecules in terms of the transport mechanism. LJ 12-6 parameters for Ti_3_C_2_O_2_ atoms (Table S1) were taken from Universal Force Field^40^ which is widely used for the parametrization of metals and novel inorganic materials, including metal-organic frameworks.^41,42^

### 2.2 Equilibration of Ti_3_C_2_O_2_-Solvent Systems

**Figure 1:**
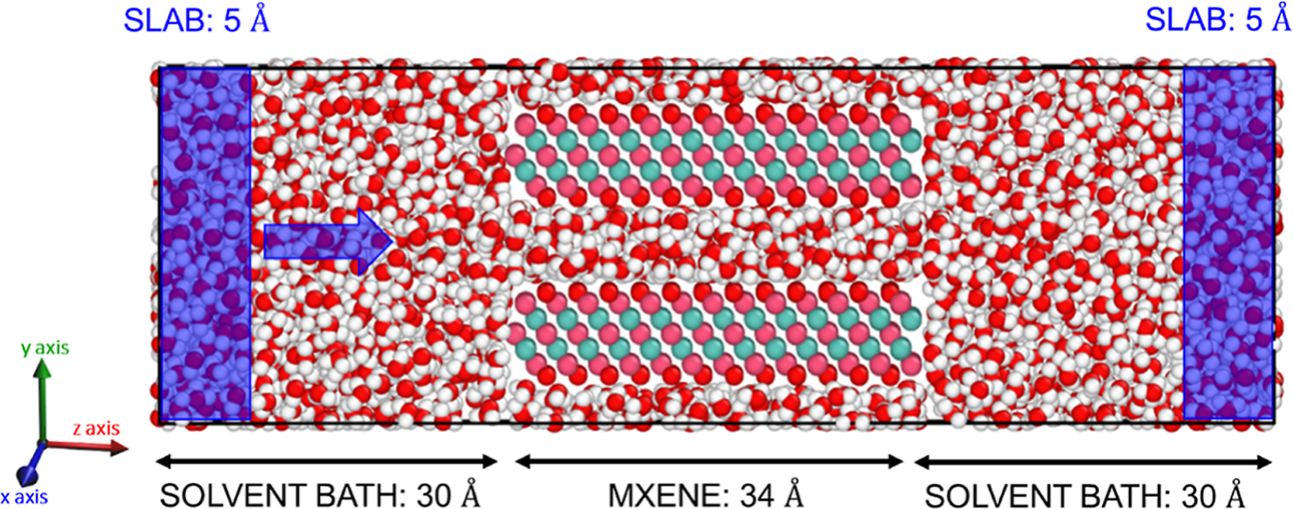
Representative simulation cell consisting of two Ti_3_C_2_O_2_ nanolayers and water molecules as solvent. Note that the system is periodic in *x*- and *y*-axes. The dimension of the solvent bath may alter after the equilibration, please see Figure S2 for solvent-specific locations.

The simulation system for each solvent was prepared by packing two solvent baths at the entrance and exit of the MXene nanochannel and filling the interlayer spacing between nanolayers with solvent molecules as depicted in Figure 1 using PACKMOL software.^34^ The Lorentz–Berthelot mixing rule was used for solvent-MXene interactions with a 14.0 Å cutoff. Electrostatic interactions were approximated by a PPPM summation. In addition, bonds and angles involving hydrogen atoms of solvent molecules were constrained by using the SHAKE algorithm. All simulations were carried out via LAMMPS,^35^ and for visualization, VMD^43^ and OVITO^44^ were used. During the minimization and equilibration steps, MXene atoms were fixed by setting the constant force acting on them to zero. Nosé–Hoover thermostat and barostat algorithms were used for the solvent molecules. Following the minimization, the volume of the system was allowed to change in only the *z*-direction (*N*_Z_*PT* ensemble). Then, 10 ps constant-volume, constant-temperature (*NVT*), and 100 ps *NPT* simulations were performed at 30 K. Finally, the system was heated to 298 K with a rate of 0.2 K ps^–1^ in the *NPT* ensemble, followed by 5 ns *NPT* at 298 K. Total volume and dimensions of the initial and equilibrated systems along with the total number of packed solvent molecules are given in Table S2 in the Supporting Information. For each solvent, four randomly packed simulation cells were generated and equilibrated.

### 2.3 NEMD Simulations of Ti_3_C_2_O_2_-Solvent Systems

In NEMD simulations, the “rigidity” of the MXene nanolayers was examined as a parameter. Therefore, in certain simulations, Ti_3_C_2_O_2_ atoms were kept completely fixed, while in others, they were “tethered” to their initial positions using a restraining force constant, which allows them to vibrate without moving. To reveal the effect of the magnitude of the restraining force, different force constants (100, 500, and 1000 kcal mol^–1^ Å^–2^) were tested. In addition, the impact of the NEMD ensemble on solvent transport was examined by employing two different ensembles, *NVT* and *NVT*_N_, where *T*_N_ refers to the temperature coupling of the nanomaterial only by using a Langevin thermostat. In *NVT*_N_ simulations, temperature coupling on solvent molecules was removed, and a constant-energy, constant-volume ensemble (*NVE*) was used for them. While applying an external force to solvent molecules, two 5 Å thick regions were selected from feed and permeate sections of the *z*-axis and defined as “force slabs” (Figure 1). A predetermined force constant was applied in the *z*-direction to solvent molecules located in these slabs. Applied pressure difference, Δ*P*, is calculated from
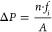
1where *n* is the number of atoms in the slab, *f*_i_ is the force constant depending on the solvent type i, and *A* is the cross-sectional area of the channel. To determine force constants resulting in a linear relation between the flux and applied pressure difference, at least three different force constants were tested for each solvent. This involved conducting independent 10 ns-long simulations with different force constants on four randomly packed replica cells (see Table S3). The number of solvent atoms passing through the MXene nanochannels during the simulation time is counted using a custom-written script (see the Supporting Information).

Finally, the tme-averaged sum over the energy of nonbonded interactions between all MXene and solvent atom pairs is divided by the time-averaged number of confined solvent atoms to compute per-atom interaction energy (referred to as *E*_int_).

## 3 Results and Discussion

### 3.1 Parameters Affecting the Flux of Confined Solvents

#### 3.1.1 Thermostating

As mentioned above, in order to reveal the effect of thermostatting strategy, simulations were performed under the *NVT* or *NVT*_N_ ensemble according to Newton’s equation of motion
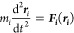
2in which *m*, ***r***, and ***F*** are the mass, position, and force acting on each atom *i*. The temperature of the system (*T*) is correlated with the velocities of atoms via
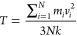
3where *k*, *N*, *m*, and *v* represent the Boltzmann constant, total number of atoms, mass, and velocity of each atom, respectively.

In EMD simulations, to control the temperature of an isothermal system, the temperature is rescaled via thermostat algorithms which are classified into two major categories: stochastic thermostats (e.g., Andersen and Langevin) and deterministic thermostats (e.g., Gaussian, Berendsen, kinetic rescaling, and Nosé–Hoover).^45,46^ In the first category of algorithms, the velocities of atoms are rescaled within a certain time step to keep the average temperature at the target value. For instance, in the Nosé–Hoover thermostat,^47,48^ the velocities are scaled by a variable (*p*_η_) relating the momentum of the heat bath (η) of mass (*Q*) via (*p*_η_ = *Q* dη/d*t*) and the instantaneous temperature of the system by coupling can be expressed as
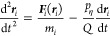
4
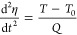
5

In the second category of algorithms, atom velocities are randomly assigned instead of scaling. For instance, in Langevin dynamics, the equation of motion is modified to include additional frictional (γ) and random forces (***R***) on the individual atoms
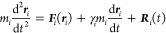
6

Collectively, applying a thermostat to a simulation system is a viable strategy to remove excess heat, avoid large fluctuations in both temperature and velocity, and improve reproducibility. Due to its deterministic nature, the Nosé–Hoover thermostat was preferred in the equilibration period where conservation of the total energy is critical. In NEMD simulations conducted under *NVT* or *NVT*_N_ ensembles, the thermostat was switched to a Langevin thermostat to more accurately model the effects of friction and random thermal motions of the particles.

The effect of different thermostats on the dynamics of various molecular systems is studied in the literature.^49^ Specifically, several studies have highlighted the critical importance of thermostat selection and thermostatting methods in simulations of confined fluids within the nanochannels or nanopores.^50−53^ For such heterogeneous systems, a thermostat can be applied to the fluid, nanoparticle, or both. The misuse of thermostats may result in unrealistic behavior of the fluid due to work being done on the system, leading to incorrect interpretations. In NEMD simulations, the use of a rigid wall model by freezing the channel atoms and reducing the computational cost was proposed as a useful strategy. However, assuming that transported fluid has an infinite thermal conductivity and the ability to continuously remove all of the excess heat is prone to severe artifacts. For a nanoconfined flow driven by a large pressure gradient, the isothermal flow assumption is prone to generate inconsistent results with the shear flow experiments.^54,55^ At this point, thermostatting the channel atoms instead of the solvent atoms has emerged as a more realistic approach. Several examples of described thermostatting strategies and their effects on the solvent transport are shown in Table 2. Ten different NEMD runs were carried out in the *NVT* ensemble with varying pressure differences for the system where the thermostat was applied to the solvent molecules and the MXene was kept rigid. Although comparable fluxes, hence an acceptable reproducibility, were obtained under similar pressure differences (see the data with superscripts a, b, c in Table 2), low solvent fluxes compared to channel-coupled thermostatting (*NVT*_N_) indicate restricted solvent dynamics, which require working under high-pressure differences. However, a considerable improvement in solvent flux was achieved for the systems where the thermostat was applied to the rigid MXene nanosheets, even at lower pressures. In NVT NEMD simulations, velocities of atoms gained by the applied external force were dampened by the thermostat when it was directly applied to the solvent molecules. Therefore, the dynamics of the system slow down, resulting in a lower flux at the same pressure, compared with the system in which the thermostat is applied only to the MXene nanosheets.

**Table 2:** Effect of Thermostating Method and Channel Wall Flexibility on the Solvent Transport^a^

ensemble	thermostating	channel wall model	restraining force constant, kcal mol^–1^ Å^–2^	pressure difference, bar	number of conducted solvent atoms, ns^–1^
*NVT*	solvent	rigid		6614	6.38
*NVT*	solvent	rigid		13,584	7.13
*NVT*	solvent	rigid		20,309	5.40
*NVT*	solvent	rigid		27,346	33.3
*NVT*	solvent	rigid		34,183	44.6^a^
*NVT*	solvent	rigid		34,739	49.3^a^
*NVT*	solvent	rigid		41,019	67.4^b^
*NVT*	solvent	rigid		41,821	65.3^b^
*NVT*	solvent	rigid		48,167	106^c^
*NVT*	solvent	rigid		48,635	116^c^
*NVT*_N_	channel	rigid		6547	10.8^d^
*NVT*_N_	channel	rigid		6948	52.0^d^
*NVT*_N_	channel	rigid		8044	14.3^e^
*NVT*_N_	channel	rigid		8498	56.2^e^
*NVT*_N_	channel	rigid		9446	10.1^f^
*NVT*_N_	channel	rigid		9976	73.3^f^
*NVT*_N_	channel	tethered	1000	6948	34.3^g^
*NVT*_N_	channel	tethered	1000	6992	35.1^g^
*NVT*_N_	channel	tethered	1000	6881	35.2^g^
*NVT*_N_	channel	tethered	500	6583	31.9^h^
*NVT*_N_	channel	tethered	500	6508	34.1^h^
*NVT*_N_	channel	tethered	500	6504	33.7^h^
*NVT*_N_	channel	tethered	100	6970	46.2^i^
*NVT*_N_	channel	tethered	100	6859	50.6^i^
*NVT*_N_	channel	tethered	100	7126	43.5^i^

a Superscripts a–i indicate simulations conducted for replica cells under similar pressure differences to examine reproducibility. Small variations in the pressure difference arise from fluctuations in the number of atoms within the force slab.

On the other hand, in NEMD simulations, imposing position restraints on the nanochannel atoms requires particular attention. When a rigid wall model with constrained atoms is used, thermostatting the channel proves to be ineffective for accurate temperature control. Since viscous heat cannot be properly dissipated, large fluctuations in temperature and velocity eventually lead to poor reproducibility and large variances in solvent flux (see data with superscripts d, e, and f in Table 2). In order to improve reproducibility, nanochannel atoms should be “tethered” to their initial positions using a suitable restraint potential instead of being completely fixed. Applying a thermostat to the tethered nanochannel atoms does not directly alter the temperature of the fluid. Instead, these atoms are employed to either add or remove energy from the fluid, ensuring that its temperature remains consistent with that of the channel. As can be seen in Figure S3 in the Supporting Information, the thermostatting with the tethering approach is robust in controlling the temperature of the fluid. Accordingly, similar water fluxes were computed at similar pressure differences when a thermostat was applied to tethered MXene atoms, regardless of the restraining force constant (see the data with superscripts g, h, and i in Table 2).

The value of the restraining force constant is also worthy of attention since wall flexibility affects the momentum exchange and friction between nanochannel and solvent atoms.^55^ Since a large force constant yields a low vibration frequency in MXene atoms, it may lead to stiffer collusions between solvent and channel atoms, hindering solvent transport. On the contrary, a low force constant allows MXene atoms to freely vibrate and exchange momentum with the solvent atoms. Nevertheless, it is important to exercise caution when determining the optimal value of the restraining force constant. Upon testing small force constants (10 and 50 kcal mol^–1^ Å^–2^), we observed significant deformation in the MXene structure (see Figure S4), indicating that force constants below 100 kcal mol^–1^ Å^–2^ were insufficient to maintain the integrity of the channel structure under high-pressure difference. On the other hand, larger force constants (500 and 1000 kcal mol^–1^ Å^–2^) lead to lower fluxes compared with that of 100 kcal mol^–1^ Å^–2^ (Table 2). We also realized that after a certain value the increase in force constant does not lead to a considerable flux alteration as implied by the similar water fluxes at 500 and 1000 kcal mol^–1^ Å^–2^. Therefore, an optimum force constant of 100 kcal mol^–1^ Å^–2^ is suggested to be used in NEMD simulations to tether the channel atoms, which agrees well with the common values used in the literature.^53,56^

#### 3.1.2 Locating the Slab Region

**Figure 2:**
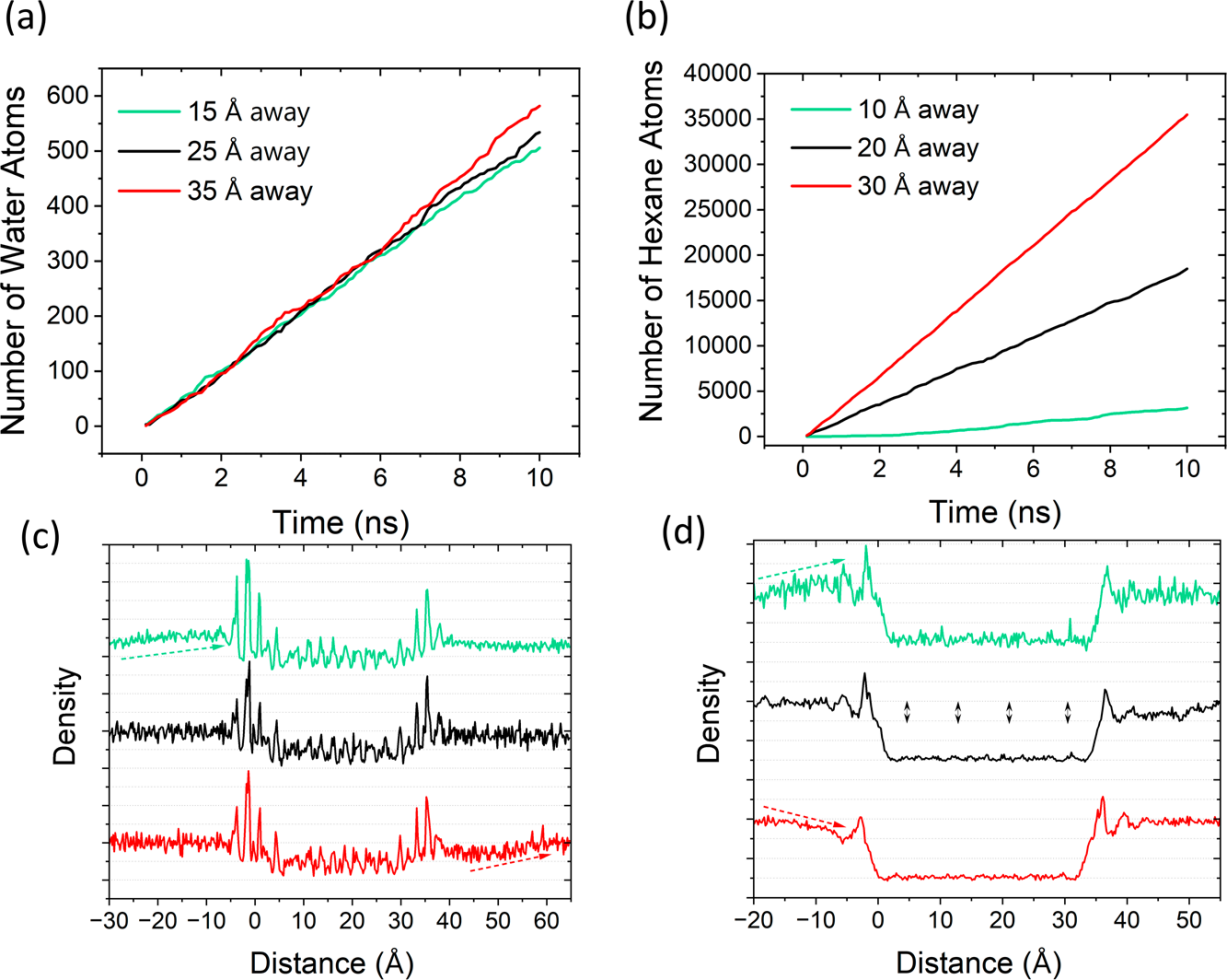
Number of (a) water and (b) hexane atoms passing through Ti_3_C_2_O_2_ nanochannels within 10 ns. Three different distances between the slab and channel entrance for a 10 Å thick slab region were tested for each solvent. Density distribution of (c) water and (d) hexane along Ti_3_C_2_O_2_ nanochannels as a function of distance. Gray dotted lines are used to guide the eye. Arrows are used to reveal the alteration in density distribution. The successive forms of (c,d) are given in the nested form in Figure S5 in Supporting Information.

Another important parameter in (BD)-NEMD simulations is the definition of the proximity of the slab region to the entrance of the nanochannel. Applying an external force to the solvent atoms located in a slab, which is sufficiently away from the mouth, should be practiced since it ensures that the flow is induced only by the density gradient between each end of the nanochannel. On the other hand, the positioning of the slab region holds little significance, as long as the solvent molecule is not too large. Water is the most commonly examined solvent in previous (BD)-NEMD studies, and since it was seen that the distance of the slab region to the nanochannel did not have a considerable influence on water flux (see Figure 2a), it has not been further evaluated. However, when (BD)-NEMD simulations involve bulky solvents such as hexane, the proximity of the slab region to the entrance of the nanochannel has a significant impact on the solvent flux, as illustrated in Figure 2b.

To define the positioning of the slab region for bulky solvents, a detectable density or concentration gradient^57^ should be observed between each end of the nanochannel in order to have a statistically acceptable solvent flux. Additionally, the curve of the density distribution at each solvent bath should be steady without ascending or descending. Although more or less similar water fluxes were observed for each location of the slab region, density distributions on the feed and permeate sides of the nanochannel are not regular and slightly ascending for the 15 and 35 Å cases (see Figure 2c). However, for the optimal distance of the slab (25 Å), there was a smooth density distribution on both sides of the nanochannel. On the contrary, the variation in density distribution is more notable for the case of hexane (see Figure 2d). Additionally, either the nanochannel entrance was not capable of providing a notable density difference or ascending or descending hexane density distribution was observed at the feed solvent bath of the system for the 15 and 35 Å cases (see Figure 2d). Therefore, the optimal distance of the slab is 20 Å for hexane. An alternative way to ensure a consistent and stable distribution of bulky solvents between the solvent baths is the expansion of the simulation system. Nevertheless, this expansion will lead to increased simulation time and higher costs. Therefore, when working with bulky solvents like hexane, it is crucial to meticulously choose the ideal distance between the slab region and channel entrance, taking into account the system’s size.

#### 3.1.3 Limiting the Slab Region

The final and equally noteworthy parameter is defining the boundaries of the slab region where the solvent atoms subjected to external force are located. (BD)-NEMD approach involves applying an external force to a predefined slab region within the bulk solvent phase,^14,25,52,57^ rather than directly applying it to all solvent molecules throughout the bulk phase.^11,23^ Mainly, an external field in the direction of the nanochannel axis is applied to the molecules in the predefined slab. The general practice is designating two slabs, each comprising at least one-third of the bulk region, positioned at either end of the channel. Accordingly, we selected two 5 Å thick slabs, located at the two ends of the simulation cell (see Figure S2 for solvent-specific location), as illustrated in Figure 1 and elaborated in Section 3.1.2. Assisted by an applied external force, molecules within this small slab region accelerate and subsequently move from the interface of two periodic bulk regions, causing an increase in the concentration in one region and a decrease in the concentration in the other.

**Figure 3:**
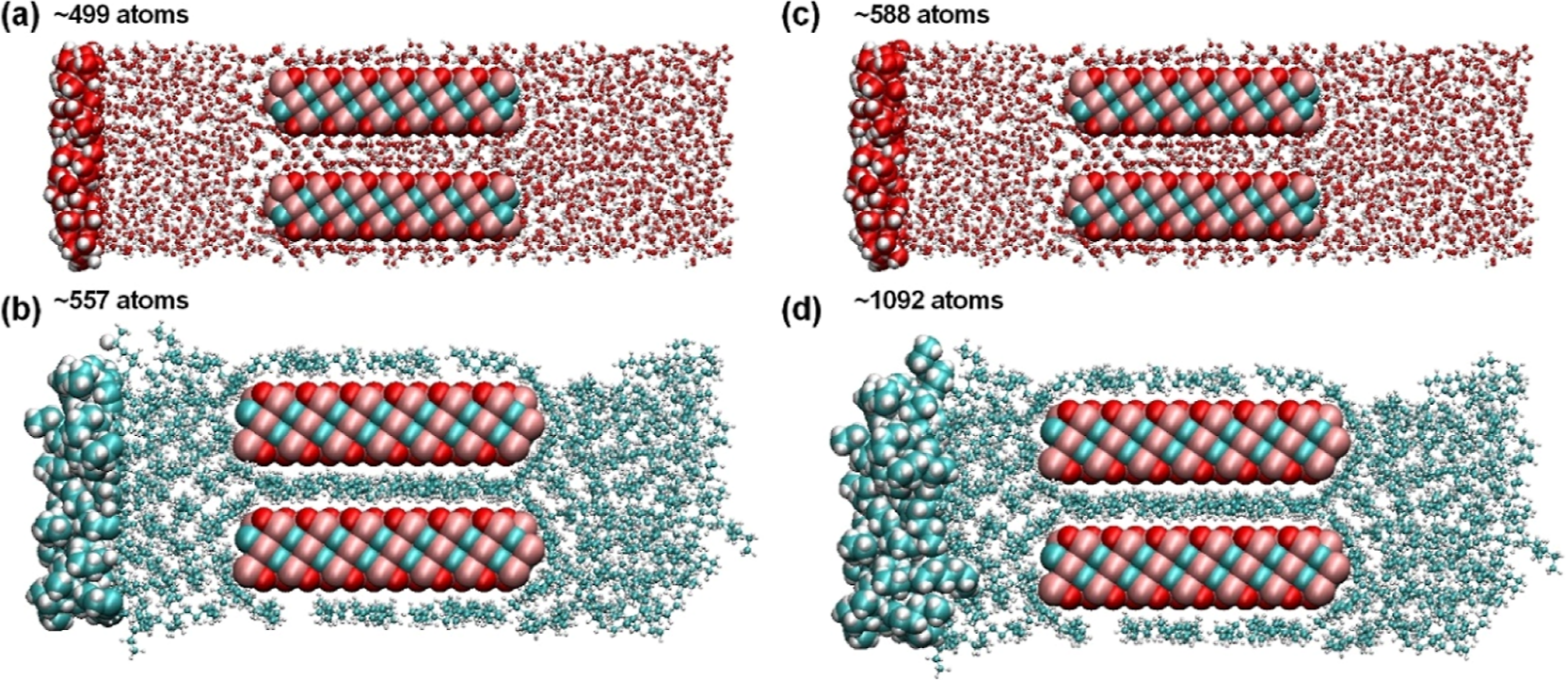
Snapshots of Ti_3_C_2_O_2_-solvent systems visualizing the relation between the size of molecule and the size of slab. Ti_3_C_2_O_2_ atoms and solvent atoms are shown in VDW and CPK representations, respectively. Solvent atoms falling within the left slab region are also shown in the VDW representation for water in (a) and hexane in (b). Other atoms of the same molecule as in-slab atoms are added to VDW representation for water (c) and hexane (d) systems. Number of atoms falling within the defined slab is given on top of the region.

For solvents with small molecular size like water, which consists of only three atoms, the definition of a slab region with sharp spatial boundaries may not pose significant challenges in the flux calculation.^14,25,52,57^ Therefore, akin to the location of the slab, the boundaries of the slab have not been further considered. However, a large solvent molecule is likely to straddle the slab boundary and may suffer from a nonuniform intramolecular force distribution. When a solvent molecule straddles the slab boundary, a few atoms of the solvent molecule are subjected to an external force, while the rest of the molecules are not. The nonuniform force distribution decreases the efficiency of the applied force and limits the overall displacement of the solvent molecule due to strong intramolecular forces such as bond, angle, torsion, and improper potentials. The effect of this anomaly becomes more apparent for large solvent molecules such as hexane and heptane compared to water (Figure 3). A notable difference in the atom count arises when all of the atoms of a straddling solvent molecule are included. As illustrated in Figure 3, the number of atoms within the specified slab increases by a mere 18% for water, whereas it surges by 96% for hexane, compared to solely including the discrete atoms within the slab’s spatial boundaries. Therefore, we modified our method to provide a whole-molecule uniform force distribution for straddling solvent molecules. In this approach, even if a single atom of the molecule falls within the slab, force is applied to all atoms of the solvent molecule. Instead of applying a constant external force (denoted by *f* in eq 1), keeping the total pressure difference constant is intended. Therefore, the total number of atoms of solvent molecules subjected to force, *n*, is counted and updated at every time step. Instead of the force constant, *f*, the multiplication, *n* × *f*, is predefined as a constant value and *f* is recalculated accordingly at every time step. Table 3 compares the water and hexane fluxes obtained by two different slab-limiting approaches. When whole-molecule uniform force distribution is intended, an apparent increase (approximately 25%) in hexane flux is obtained. Conversely, the water flux remains largely unaffected by this strategy, with the observed deviation falling within the range of statistical error. Moreover, we anticipate that this approach is well-suited for NEMD simulations involving mixtures of molecules with varying molecular masses. For instance, in the case of an aqueous solution containing solutes such as dyes or pharmaceuticals with molecular masses greater than that of water, buoyancy effects and artifacts resulting from nonuniform force distribution may impact the calculation of the flux.^57^ Therefore, the whole-molecule uniform force distribution approach ensures acceleration of both solvent and solute molecules. Finally, this approach allows us to carry out NEMD simulations at a constant pressure difference without concern for variations in the number of atoms within the slab region; hence, it provides an opportunity to directly compare the flux of different solvents under an identical pressure difference, as demonstrated in Table 3.

**Table 3:** Water and Hexane Fluxes Obtained by Two Different Slab-Limiting Approaches

solvent	force distribution	pressure gradient, bar	number of conducted solvent atoms, ns^–1^
water	only in-slab	13,364	60.5 ± 3.27
hexane	only in-slab	15,158	30.3 ± 6.75
water	whole-molecule	14,846	63.7 ± 4.18
hexane	whole-molecule	14,846	39.8 ± 5.04

### 3.2 Extrapolating Computed Flux to Experimentally Related Pressure Gradients

**Figure 4:**
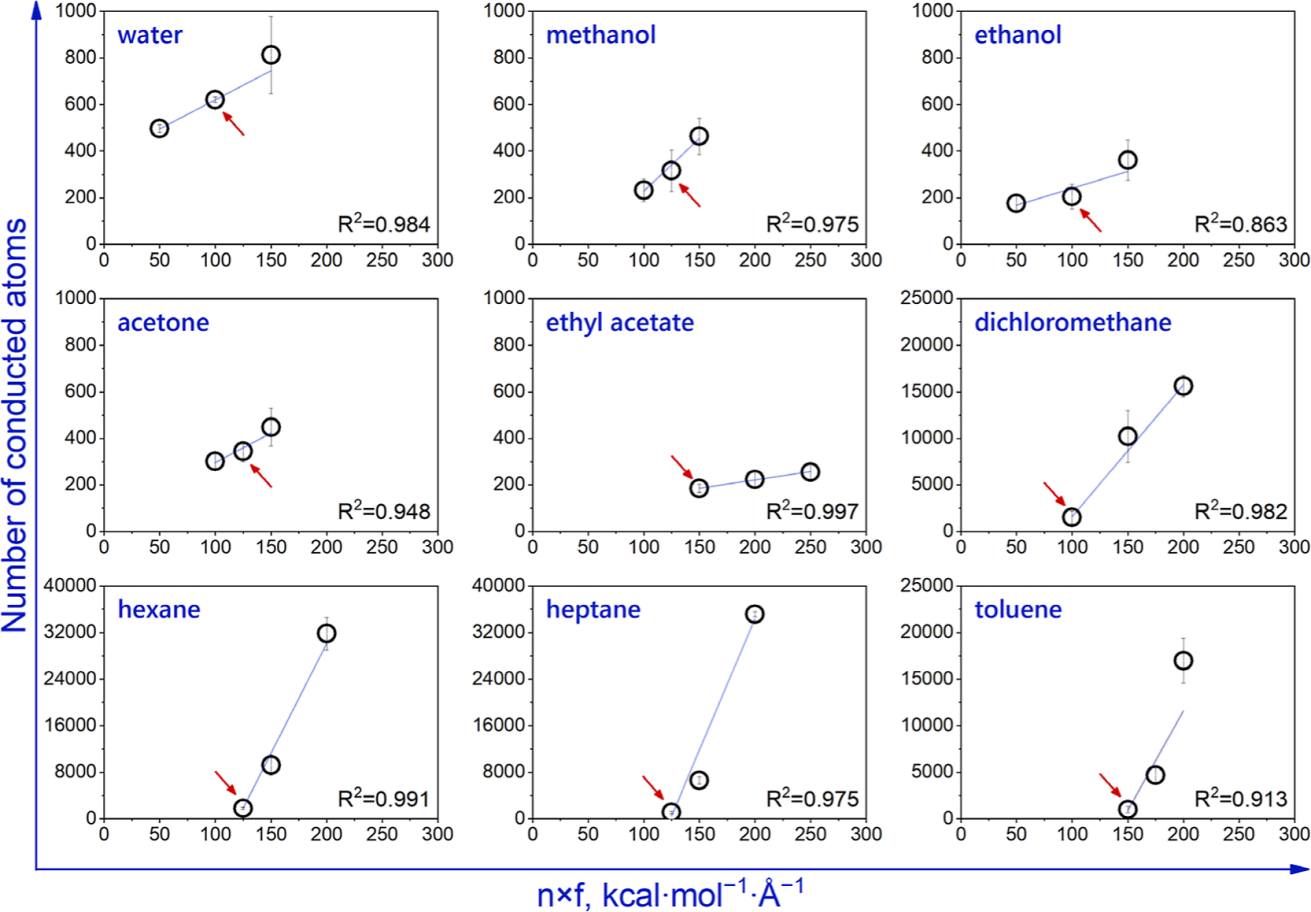
Three *n* × *f* values, which give an approximately linear relationship (*R*^2^ > 0.85) between *n* × *f* and the number of conducted atoms for each solvent. *n* × *f* minimizing the standard deviation and used to calculate permeance is marked with a red arrow. Error bars are calculated over four discrete matrices.

Time correlation functions in equilibrium MD simulations are subjected to significant statistical error since they represent the average responses to naturally occurring, relatively small fluctuations. Therefore, equilibrium MD simulations are inadequate to mimic solvent transport in pressure-driven membrane filtration processes. However, as the computational counterpart of real-world filtration tests, NEMD simulations benefit from a high-pressure difference to generate statistically significant data within a reasonable simulation time frame. It is noteworthy that the applied artificial external force in NEMD simulations leads to large pressure differences, which are significantly higher compared with filtration experiments. The reason for applying such high-pressure differences is to ensure a reasonable signal-to-noise ratio through large perturbations. However, the question of determining the optimal magnitude, whether it is high or low, is worthy of attention. Unfortunately, there is no recipe for the precise magnitude of this force since it depends on the solvent transport properties and intrinsic permeability of the channel. To be able to extrapolate solvent flux to the experimentally related pressures, the relation between the applied pressure difference and the number of solvent atoms conducted by the channel should be linear. We tested our optimized NEMD method for nine solvents and determined *n* × *f* values showing a linear correlation with the solvent flux. For each solvent, four different simulation cells were generated via random packing and then were equilibrated. Finally, each cell was simulated under identical conditions to determine the average flux. The pressure gradient was varied by changing the *n* × *f* values between 50 and 250 kcal mol^–1^ Å^–1^. Raw data and associated standard deviations are given in Table S3. In this way, we were able to identify three *n* × *f* values which give an approximately linear relationship (*R*^2^ > 0.85) between the applied pressure difference and the number of conducted atoms for each solvent (Figure 4). We would like to emphasize that there are significant variations in the range of *n* × *f* values falling within the linear region depending on the solvent properties. To illustrate, for water, *n* × *f* values of 50, 100, and 150 kcal mol^–1^ Å^–1^ give a correlation with *R*^2^ = 0.98, indicating that even 50 kcal mol^–1^ Å^–1^ is sufficient to extrapolate the obtained permeance to experimentally related pressure gradients. On the contrary, for ethyl acetate, the numbers of conducted atoms calculated for *n* × *f* values of 100 and 150 kcal mol^–1^ Å^–1^ are almost equal. To obtain a linear correlation (*R*^2^ = 0.99), higher *n* × *f* values (150, 200, and 250 kcal mol^–1^ Å^–1^) are required due to the low permeability of this solvent. Therefore, we propose that the optimal pressure difference may be specific to the solvent-channel system, and it is essential to perform this analysis for each solvent. Finally, *n* × *f* value minimizing the standard deviation was chosen from the linear region and used to calculate the solvent permeance of the channel as follows
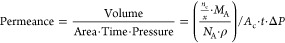
7where *n*_c_ is the number of atoms conducted by the nanochannel, *x* is the number of atoms in one solvent molecule, *M*_A_ is the molecular mass of solvent, *N*_A_ is Avogadro’s number, ρ is the solvent density, *A*_c_ is the cross-sectional area of the nanochannel where solvent transport takes place, *t* is the duration of NEMD run, and Δ*P* is the pressure difference defined in eq 1.

**Figure 5:**
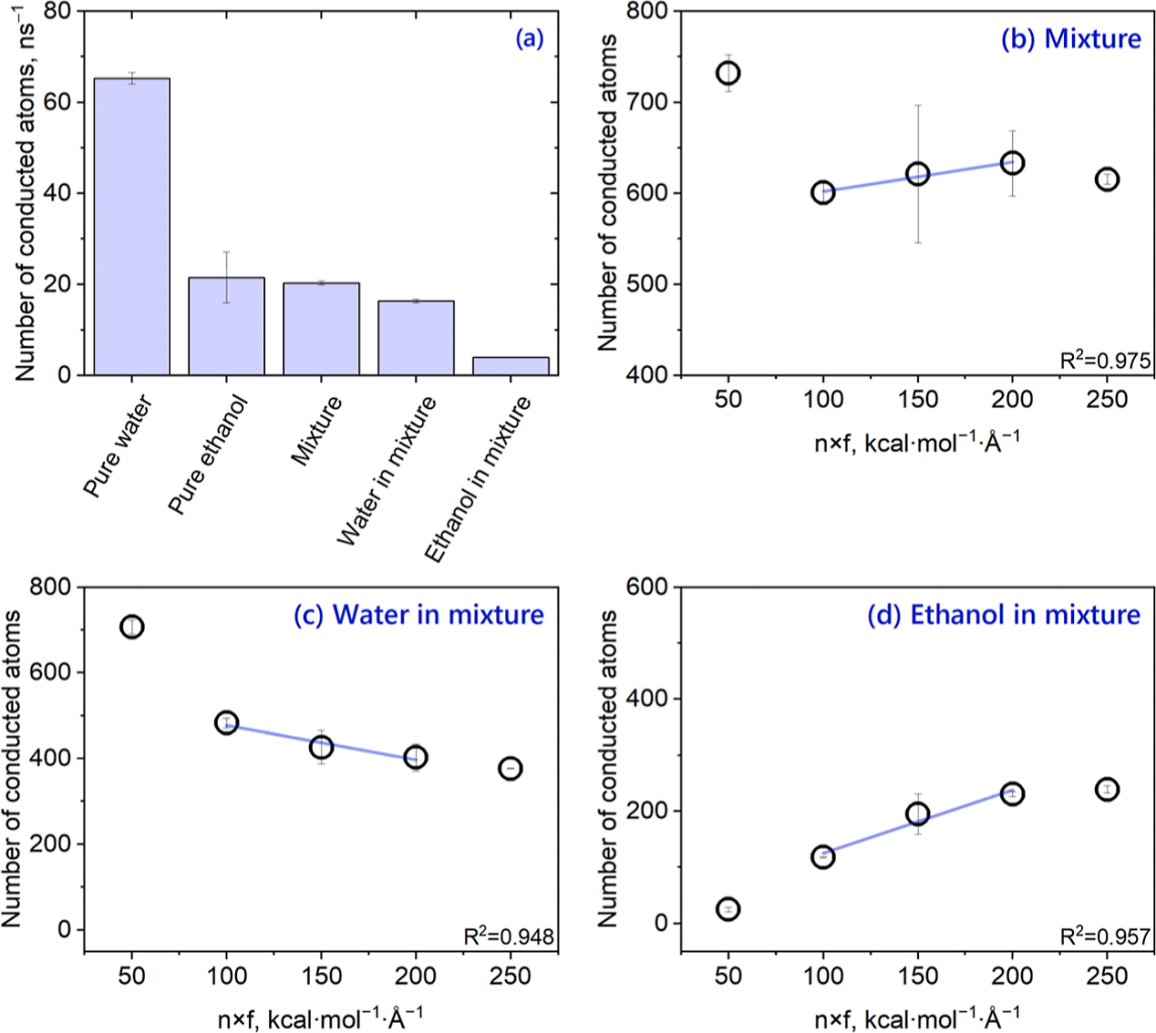
NEMD simulations of a 50–50% water–ethanol mixture. (a) Comparison between the flux of pure solvents, mixture, and its components at the *n* × *f* value of 100. (b) Relation between *n* × *f* values and the flux of (b) water–ethanol mixture, (c) water in the mixture, and (d) ethanol in the mixture. 30 ns-long NEMD simulations were performed for the case of the water–ethanol mixture, and error bars are calculated over four discrete matrices.

We also tested our simulation protocol for a mixture containing 50% ethanol and 50% water. At the *n* × *f* value of 100, the flux of the mixture was found to be close to that of pure ethanol (Figure 5a), implying that the transport of the mixture is governed by its slowest component. This behavior of water–ethanol mixtures has also been observed in previous studies and was attributed to the coupling of ethanol molecules to water molecules through hydrogen bonding interactions, which hinders the transport of water.^58^ From the NEMD simulations of pure solvents, *n* × *f* values falling within the linear region had been determined as 50, 100, and 150 for both water and ethanol (Figure 4). However, for the water/ethanol mixture, these values failed to provide a linear relation between the pressure gradient and solvent flux. Thus, *n* × *f* values governing the linear region for the water–ethanol mixture were found to be 100, 150, and 200 based on the conduction of individual components and the mixture (Figure 5b–d). This difference between pure solvents and their mixtures supports our suggestion that obtaining statistically satisfying flux data strongly depends on the specific transport properties of a solvent-channel system and the (BD)-NEMD protocol should be optimized accordingly.

### 3.3 Correlation between Solvent Properties and Calculated Permeance

**Figure 6:**
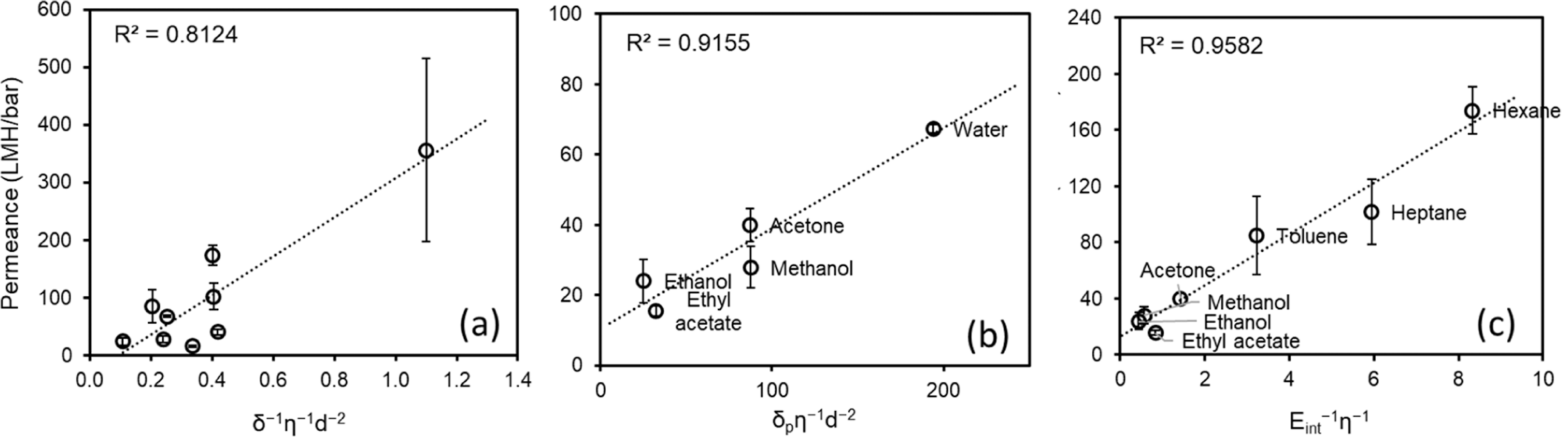
Relation between solvent permeance and the combination of solvent properties as (a) δ^–1^η^–1^*d*^–2^, (b) δ_p_η^–1^*d*^–2^, and (c) *E*_int_^–1^η^–1^. Relevant solvent properties are provided in Table S4 (LMH/bar: L m^–2^ h^–1^ bar^–1^).

While the correlation between solvent properties and permeance is significantly influenced by both the intrinsic solvent permeability of the nanochannel and nanochannel-solvent interactions, several experimental studies employing 2D nanochannels have been able to report an approximately linear relationship. In these studies, viscosity (η) alone^59,60^ or a combined parameter^61−63^ comprising viscosity, Hansen solubility parameter (δ), polar term of Hansen solubility parameter (δ_p_), and kinetic diameter (*d*) were correlated with solvent permeance. In the literature, various forms of combined parameters are encountered and tailored to suit the transport mechanism of the studied channel. To validate our suggested methodology, we correlated our simulated permeance data with various combined parameters in Figure 6. With a commonly used combined parameter (δ^–1^η^–1^*d*^–2^), a correlation coefficient of *R*^2^ = 0.81 was obtained when all studied solvents were included in the analysis (Figure 6a). For this correlation, all terms of Hansen solubility parameter [dispersion (δ_D_), polar (δ_P_), and hydrogen bonding (δ_H_)] were accounted. On the other hand, a higher correlation coefficient (*R*^2^ = 0.92) was achieved (see Figure 6b) when only the polar term of the Hansen solubility parameter was used in the numerator of the equation for the combined parameter (δ_p_η^–1^*d*^–2^). Given that, only the polar term of the Hansen solubility parameter is considered in this analysis, solvents with the lowest polarity, such as toluene (0.099), *n*-heptane (0.012), and *n*-hexane (0.009) according to the Reichard *t* index,^64^ were excluded. In addition to the experimentally related macroscopic solvent properties, we propose a new combined parameter based on a simulation-derived property, the interaction energy between confined solvent molecules and MXene nanosheets (*E*_int_). When the interaction energy per confined solvent atom and viscosity of the solvent are included in the denominator of the combined parameter (*E*_int_^–1^η^–1^), a remarkably linear correlation (*R*^2^ = 0.96) was achieved by our suggested methodology (Figure 6c). This correlation implies that strong interactions between the solvent and channel lead to increased transport resistance and decreased permeability, aligning with the experimental reports.^65^

### 3.4 Comparison to Experimental Solvent Permeance of MXene Nanolaminates

**Figure 7:**
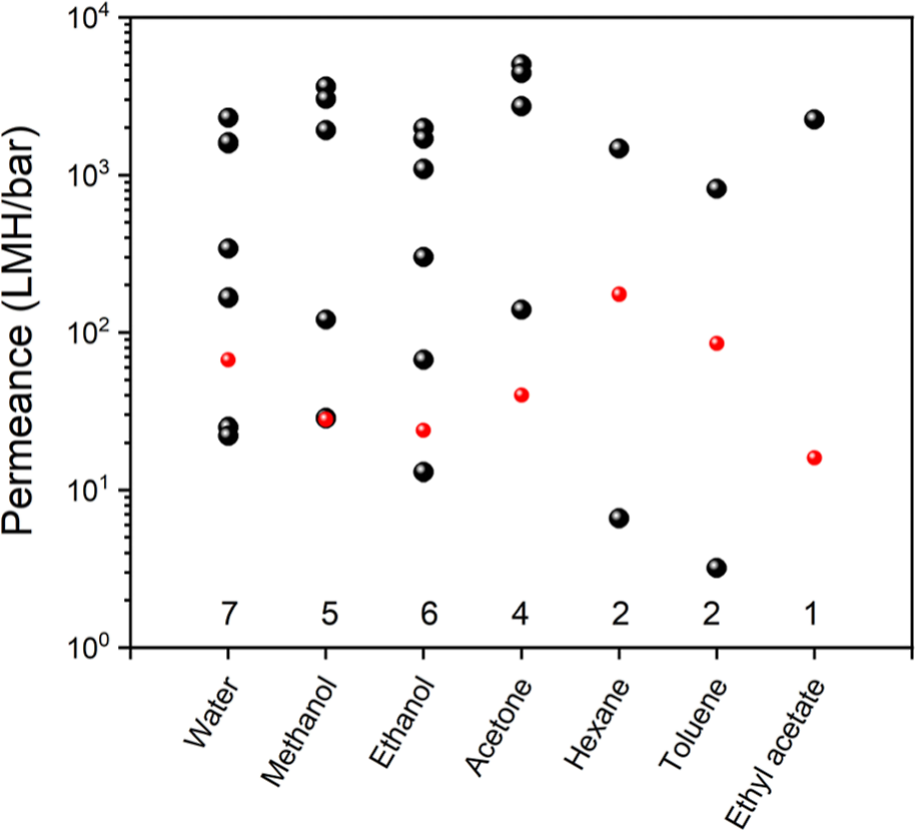
Comparison of the permeances of various organic solvents calculated by our approach with the experimentally measured permeances of MXene nanolaminates in the literature. Numbers on the top of solvent names represent the total number of experimental data. Our simulation data are shown in red. The literature data for MXene nanolaminates are provided in Table S5 in the Supporting Information.

Finally, we compare the simulated solvent permeances with the previous experimental findings on MXene nanolaminates to validate our methodology (Figure 7). We attribute large variations in experimental solvent permeances to differences in membrane fabrication and testing procedures (details are given in Table S5). Even a small variation in the interlayer distance, which is challenging to precisely control, may have a significant effect on solvent permeance. Moreover, parameters such as lateral flake size and membrane thickness affect the transport resistance by altering tortuosity and defect formation. Testing conditions, such as filtration setup (dead-end or cross-flow filtration systems) or applied pressure difference, are also known to be critical factors in determining the performance of nanolaminates. We emphasize that our simulations represent an ideal system where the only intrinsic permeability of MXene nanochannels and MXene-solvent interactions are accounted for, while the effects of defects and swelling are excluded. Even so, the solvent permeances calculated using our approach fall within the range of experimentally measured values, suggesting that the developed methodology is suitable for simulating pressure-driven filtration experiments involving 2D nanolaminates and a diverse range of solvents.

## 4 Conclusions

Molecular simulation is a powerful tool to predict the intrinsic permeance of existing or theoretical membranes, excluding the inherent variability introduced during membrane fabrication or testing. The (BD)-NEMD method has been widely utilized to examine the transport of water through nanochannels; however, for bulky solvents, methodological details require refinement. In this work, we offer a guide for (BD)-NEMD simulations of organic solvent transport in nanochannels. We provided a step-by-step optimization of the (BD)-NEMD protocol to avoid neglecting the effect of various parameters that may be disregarded for water but warrant thorough consideration for bulky solvents. We employed a recently popular 2D material, MXene (Ti_3_C_2_O_2_) nanochannel, as a model membrane and computed the permeance of methanol, ethanol, acetone, *n*-hexane, *n*-heptane, toluene, ethyl acetate, dichloromethane, and water. First, we evaluated the effect of the thermostatting method in terms of transport resistance and reproducibility. The tethered channel wall model, with an optimum restraining force constant of 100 kcal mol^–1^ Å^–2^, results in minimum interference with the dynamics of the system, as well as improved temperature control and reproducibility. Next, we showed that the distance of the force slab from the channel entrance influences the permeance of bulky solvents. Thus, it is important to carefully designate the location of the slab to ensure a steady density distribution on both the feed and permeate sides. Defining the slab boundaries is also worthy of attention since bulky solvent molecules straddling the spatial boundaries cause nonuniform intramolecular force distribution, interfering with the acceleration of the molecule. Therefore, we modified our method to provide a whole-molecule uniform force distribution and keep the total pressure difference (*n* × *f*) constant, instead of external force (*f*). Furthermore, in order to extrapolate the calculated flux to experimentally related pressure gradients, a linear relation between the pressure gradient and the solvent flux was obtained. Accordingly, we determined three magnitudes of the pressure difference correlated with the flux for each solvent. Calculated solvent permeances were then correlated with solvent properties (e.g., viscosity, Hansen solubility parameter, kinetic diameter, and interaction energy). We obtained a notably linear correlation with an *R*^2^ value of 0.96 when the combined parameter of viscosity and a simulation-derived property, interaction energy, is taken into account. Lastly, we demonstrated that the calculated solvent permeances are in line with the experimentally reported data on MXene-based nanolaminate membranes. We anticipate that our new optimized methodology will enable more accurate simulations of membrane transport across a wide range of solvents and membrane materials. Such predictive efforts potentially lead to new avenues in the OSN and mitigate the environmental burden of separations in organic media on the way to a greener industry.

## Supporting Information Available

Total number of data having the corresponding % relative error to experimental density; partial atomic charge distribution for the primitive cell of Ti_3_C_2_O_2_; temperature of the MXene nanochannel and solvent (water) as a function of time; side view of MXene nanolayers which are tethered with a spring constant; density distributions of water and hexane along Ti_3_C_2_O_2_ nanochannels as a function of distance; LJ 12-6 parameters for MXene atoms; total volume and dimensions of the initial and equilibrated systems; raw flux data and associated standard deviations calculated by changing the *n* × *f*; relevant solvent properties; and experimentally measured solvent permeances of MXene nanolaminates from the literature (PDF)
LAMMPS script file including parameters and procedure, specific for (BD)-NEMD simulations along with the corresponding data file, Python script for solvent flux calculation, and corresponding input file observed from LAMMPS (BD)-NEMD simulations (ZIP)
