# Supplementary material for: Guide for Nonequilibrium Molecular Dynamics Simulations of Organic Solvent Transport in Nanopores: The Case of 2D MXene Membranes
Source: J Chem Theory Comput. 2024 Nov 4;20(21):9642–54. doi: 10.1021/acs.jctc.4c00693 (PMC11562068; doi:10.1021/acs.jctc.4c00693)
Supplement: Supplementary file 1 — ct4c00693_si_001.pdf [file ct4c00693_si_001.pdf]

Supporting Information

Guide for Nonequilibrium Molecular Dynamics Simulations of Organic Solvent Transport in Nanopores: The Case of 2D MXene Membranes

Aysa Güvensoy-Morkoyun[1], Tuğba Baysal[2],

Ş. Birgül Tantekin-Ersolmaz[1,3,*], Sadiye Velioğlu[2,4,*]

[1]Department of Chemical Engineering, Istanbul Technical University, Maslak, Istanbul, 34469, Türkiye.

[2]Institute of Nanotechnology, Gebze Technical University, Gebze, Kocaeli, 41400, Türkiye.

[3]Synthetic Fuels & Chemicals Technology Center (SENTEK), Istanbul Technical University, Maslak, Istanbul, 34469, Türkiye.

[4]Nanotechnology Research Center (NUAM), Gebze Technical University, Gebze, Kocaeli, 41400, Türkiye.

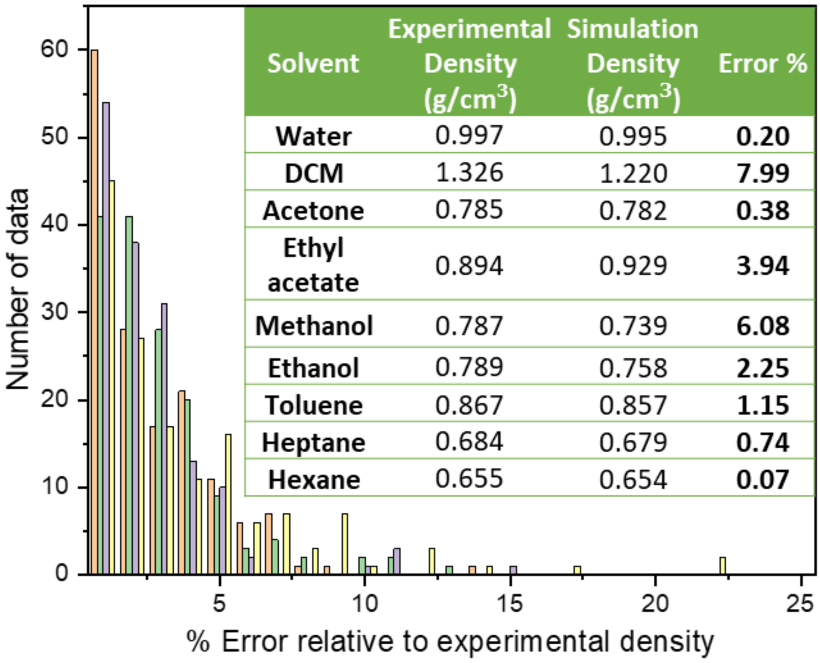

Figure S1. The total number of data having the corresponding % relative error to experimental density. They were calculated over 153 solvents by the study of Dodda *et al.*[1]

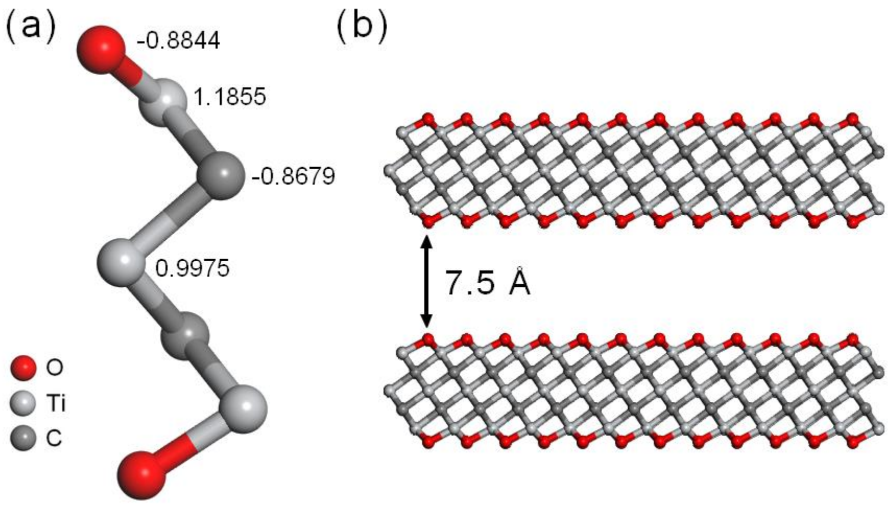

Figure S2. Model of $Ti_3C_2O_2$ **(a)** Partial atomic charge distribution for the primitive cell of $Ti_3C_2O_2$, **(b)** A supercell having two $Ti_3C_2O_2$ nanolayers with dimensions of 30×30×34 Å^3 and separated by an interlayer distance of 7.5 □.

Table S1. LJ 12-6 parameters for MXene atoms taken from UFF force field.[2]

Atom	ε, kcal mol^{-1}	σ, Å
Ti	0.0170	2.8286
C	0.1051	3.4309
O	0.0600	3.1181

Table S2. Total number of packed solvent atoms and molecules as well as volume and dimensions of the equilibrated simulation cells. Note that all solvents were initially packed into a simulation box having dimensions of 30×30×94 □ and a volume of 84600 □^{3}.

Solvent	Number of solvent atoms/molecules	Equilibrated Dimension (□)	Equilibrated Volume (□^{3})
Water			
Cell1	6942/2314	30×30×94.4	84717
Cell2	6942/2314	30×30×95.7	86130
Cell3	6942/2314	30×30×95.7	86130
Cell4	6942/2314	30×30×95.7	86130
Dichloromethane			
Cell1	3455/691	30×30×88.9	80037
Cell2	3455/691	30×30×91.1	82031
Cell3	3455/691	30×30×88.9	80046
Cell4	3455/691	30×30×83.6	75217
Acetone			
Cell1	5690/569	30×30×81.4	73237
Cell2	5690/569	30×30×82.2	73947
Cell3	5690/569	30×30×82.0	73812
Cell4	5690/569	30×30×81.5	73262
Ethyl acetate			
Cell1	5978/427	30×30×82.0	74281
Cell2	5978/427	30×30×84.2	75780
Cell3	5978/427	30×30×83.6	75240
Cell4	5978/427	30×30×83.9	75510
Methanol			
Cell1	6204/1034	30×30×83.7	75397
Cell2	6204/1034	30×30×84.8	76356
Cell3	6204/1034	30×30×86.1	77526
Cell4	6204/1034	30×30×86.1	77526
Ethanol			
Cell1	6444/716	30×30×84.3	75783
Cell2	6444/716	30×30×85.8	77112
Cell3	6444/716	30×30×85.6	76921
Cell4	6444/716	30×30×83.2	74870
Toluene			
Cell1	5925/395	30×30×80.5	72555
Cell2	5925/395	30×30×84.0	75600
Cell3	5925/395	30×30×84.2	75798
Cell4	5925/395	30×30×84.0	75600
Heptane			
Cell1	6256/272	30×30×78.2	70266
Cell2	6256/272	30×30×78.7	70812
Cell3	6256/272	30×30×78.5	70668
Cell4	6256/272	30×30×78.7	70812
Hexane			
Cell1	6040/302	30×30×78.0	70091
Cell2	6040/302	30×30×76.5	68850
Cell3	6040/302	30×30×76.4	68751
Cell4	6040/302	30×30×76.5	68868

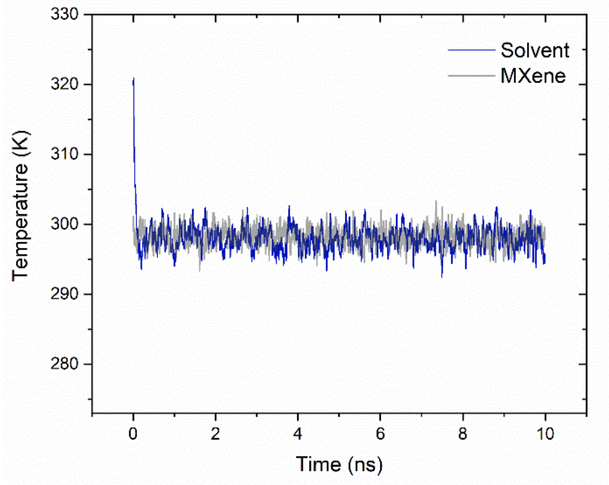

Figure S3. The temperature of MXene nanochannel and solvent (water) as a function of time. For this system, the thermostat is applied to the MXene nanochannel, and the MXene atoms are tethered with a restraining force constant of 100 kcal mol^{-1} $Å^{-2}$.

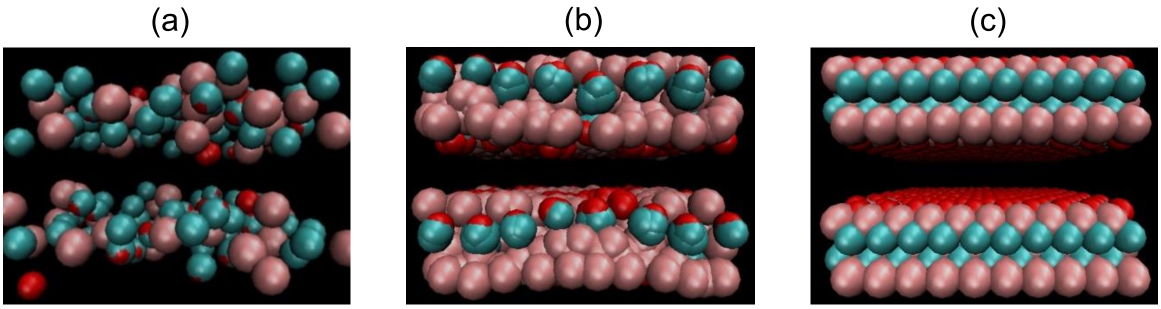

Figure S4. Side view of MXene nanolayers which are tethered with a spring constant of (a) 10, (b) 50, and (c) 500 kcal mol^{-1} $Å^{-2}$.

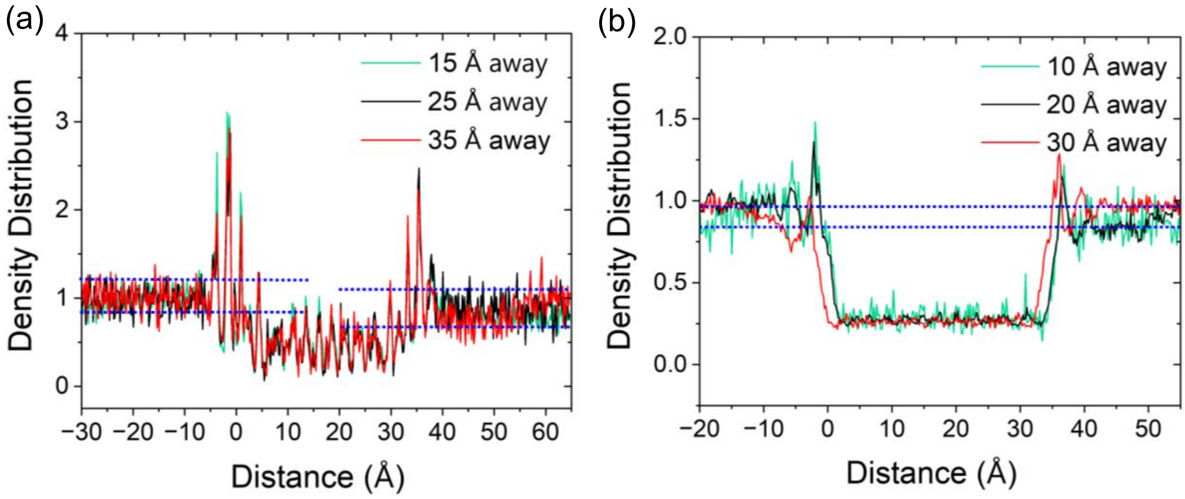

Figure S5. Density distribution of (a) water and (b) hexane along $Ti_3C_2O_2$ nanochannels as a function of distance. Blue dotted lines are used to reveal the average density difference between each solvent bath.

Table S3. Raw flux data and associated standard deviations calculated by changing the *n*×*f* values between 50 and 250 kcal mol^{-1} □$^{-1}$. The flux data is obtained from 10-ns-long NEMD simulations. Three *n*×*f* values giving a linear relationship between applied pressure and the number of conducted atoms are shown with related R^2. The *n*×*f* value chosen to calculate the flux is shown in red.

Solvent	n×f, kcal mol^{-1} □$^{-1}$	Pressure, bar	Number of conducted atoms	The average number of conducted atoms	Standard deviation	R^2
Water	50	7423	499	497	17.67	0.984
Water	50	7423	499			
Water	50	7423	517			
Water	50	7423	474			
Water	100	14846	605	620	11.94	
Water	100	14846	625			
Water	100	14846	617			
Water	100	14846	633			
Water	150	22269	976	813	166.00	
Water	150	22269	738			
Water	150	22269	921			
Water	150	22269	616			
Water	200	29692	997	1203	274.34	
Water	200	29692	1478			
Water	200	29692	1398			
Water	200	29692	939			
Water	250	37115	2218	1905	479.63	
Water	250	37115	2410			
Water	250	37115	1532			
Water	250	37115	1460			
Hexane	100	14846	383	356	76.15	
Hexane	100	14846	452			
Hexane	100	14846	297			
Hexane	100	14846	293			
Hexane	125	18557	2030	1833	179.72	0.991
Hexane	125	18557	1791			
Hexane	125	18557	1678			
Hexane	150	22269	11119	9240	1744.41	
Hexane	150	22269	8737			
Hexane	150	22269	10035			
Hexane	150	22269	7069			
Hexane	200	29692	30096	31816	2795.87	
Hexane	200	29692	31914			
Hexane	200	29692	29530			
Hexane	200	29692	35722			
Ethyl acetate	100	14846	181	187	16.90	
Ethyl acetate	100	14846	170			
Ethyl acetate	100	14846	185			
Ethyl acetate	100	14846	210			
Ethyl acetate	150	22269	187	185	18.42	0.997
Ethyl acetate	150	22269	184			
Ethyl acetate	150	22269	207			
Ethyl acetate	150	22269	162			
Ethyl acetate	200	29692	218	224	35.80	
Ethyl acetate	200	29692	249			
Ethyl acetate	200	29692	252			
Ethyl acetate	200	29692	175			

Ethyl acetate	250	37115	281	256	41.49	
Ethyl acetate	250	37115	284			
Ethyl acetate	250	37115	262			
Ethyl acetate	250	37115	195			
Heptane	100	14846	215	212	57.56	
Heptane	100	14846	152			
Heptane	100	14846	289			
Heptane	100	14846	192			
Heptane	125	18557	1053	1105	251.62	
Heptane	125	18557	884			
Heptane	125	18557	1379			
Heptane	125	18557	372			
Heptane	150	22269	6929	6621	598.70	0.967
Heptane	150	22269	7003			
Heptane	150	22269	5931			
Heptane	150	22269	2853			
Heptane	200	29692	34687	35158	414.84	
Heptane	200	29692	35320			
Heptane	200	29692	35468			
Heptane	200	29692	30127			
Methanol	100	14846	197	233	48.00	
Methanol	100	14846	284			
Methanol	100	14846	263			
Methanol	100	14846	187			
Methanol	125	18557	370	316	89.01	0.975
Methanol	125	18557	236			
Methanol	125	18557	413			
Methanol	125	18557	245			
Methanol	150	22269	454	464	77.84	
Methanol	150	22269	572			
Methanol	150	22269	387			
Methanol	150	22269	442			
Methanol	175	25980	1053	1117	95.30	
Methanol	175	25980	1257			
Methanol	175	25980	1094			
Methanol	175	25980	1062			
Methanol	200	29692	2589	2894	725.47	
Methanol	200	29692	3825			
Methanol	200	29692	3041			
Methanol	200	29692	2121			
Toluene	100	14846	180	195	75.24	
Toluene	100	14846	95			
Toluene	100	14846	261			
Toluene	100	14846	244			
Toluene	125	18557	211	180	33.89	
Toluene	125	18557	136			
Toluene	125	18557	172			
Toluene	125	18557	202			
Toluene	150	22269	1029	997	328.17	
Toluene	150	22269	1308			
Toluene	150	22269	441			
Toluene	150	22269	654			
Toluene	175	25980	4921	4702	913.70	0.913
Toluene	175	25980	5191			
Toluene	175	25980	5339			
Toluene	175	25980	3356			

Toluene	200	29692	18066	16982	2418.49	
Toluene	200	29692	18795			
Toluene	200	29692	17640			
Toluene	200	29692	13425			
Ethanol	50	7423	133	176	34.16	0.851
Ethanol	50	7423	166			
Ethanol	50	7423	212			
Ethanol	50	7423	192			
Ethanol	100	14846	136	205	53.15	
Ethanol	100	14846	190			
Ethanol	100	14846	238			
Ethanol	100	14846	254			
Ethanol	150	22269	239	362	86.13	
Ethanol	150	22269	409			
Ethanol	150	22269	366			
Ethanol	150	22269	432			
Ethanol	200	29692	445	1340	316.21	
Ethanol	200	29692	1003			
Ethanol	200	29692	1388			
Ethanol	200	29692	1630			
Acetone	50	7423	368	309	42.30	0.948
Acetone	50	7423	306			
Acetone	50	7423	295			
Acetone	50	7423	268			
Acetone	100	14846	343	301	34.30	
Acetone	100	14846	259			
Acetone	100	14846	301			
Acetone	100	14846	302			
Acetone	125	18557	335	345	43.64	
Acetone	125	18557	292			
Acetone	125	18557	397			
Acetone	125	18557	355			
Acetone	150	22269	480	448	80.35	
Acetone	150	22269	338			
Acetone	150	22269	527			
Acetone	150	22269	447			
Acetone	200	29692	2102	2662	504.63	
Acetone	200	29692	2368			
Acetone	200	29692	3081			
Acetone	200	29692	3096			
Dichloromethane	50	7423	200	290	91.89	0.982
Dichloromethane	50	7423	279			
Dichloromethane	50	7423	418			
Dichloromethane	50	7423	263			
Dichloromethane	100	14846	950	1533	684.18	
Dichloromethane	100	14846	1347			
Dichloromethane	100	14846	2523			
Dichloromethane	100	14846	1310			
Dichloromethane	150	22269	6948	10210	2795.67	
Dichloromethane	150	22269	10425			
Dichloromethane	150	22269	13748			
Dichloromethane	150	22269	9717			
Dichloromethane	200	29692	17362	15615	1167.14	
Dichloromethane	200	29692	15127			
Dichloromethane	200	29692	14958			
Dichloromethane	200	29692	15011			

Table S4. Relevant solvent properties used to calculate combined parameters. Time-averaged sum over the energy of non-bonded interactions between all MXene and solvent atom pairs is divided by the time-averaged number of confined solvent atoms to compute per-atom interaction energy (referred to as E_{int}).

Solvent	Viscosity[3], η, mPa s	Hansen solubility[6], δ, $MPa^{0.5}$	Kinetic diameter[6], nm	Hansen, intermolecular forces, δp, $MPa^{0.5}$	MXene-Solvent interaction (E_{int}), kcal mol^{-1} $atom^{-1}$	Permeance, L m^{-2} h^{-1} bar^{-1}
Dichloromethane	0.413[4]	20.2[7]	0.33[8]	6.3	4.805	356
Water	0.916	47.9	0.3	16	11.766	67
Methanol	0.540	29.7	0.51	12.3	3.290	28
Acetone	0.310	20.0	0.62	10.4	2.268	40
Ethanol	1.080	26.6	0.57	8.8	2.133	24
Ethyl acetate	0.426	18.2	0.62	5.3	2.821	16
Toluene	0.550	18.2	0.7	1.4	0.563	85
Hexane	0.297	14.9	0.75	0.1	0.404	174
Heptane	0.420[5]	15.3[7]	0.62[9]	0.1	0.400	102

Table S5. Experimentally measured solvent permeances of MXene nanolaminates in the literature.

Ref.	Interlayer distance (nm)	Lateral flake size (nm)	Membrane thickness (nm)	Pressure (bar)	Pure solvent permeance (LMH/bar)						
					Water	Methanol	Ethanol	Acetone	Hexane	Toluene	Ethyl acetate
Kang, 2017[10]	0.45	200	90	5.0	25				6.6	3.2	
Li, 2020[11]	0.35	2500	500	1.0	340		300				
Shao, 2021[12]	0.39	450	496	3.0	22.09	28.5	13				
Wang, 2018[13]	2.00	3000	230	1.0	2302	3629	1984	5022.4			
Wu, 2019[6]	0.38	3000	320	1.0	1578	3037	1696	2719	1468	818	2249
Wei,2019[14]	0.43	500	140	0.5	1606	1917	1088	4430			
Xing, 2020[15]	0.55	500	400	1.0	166	121	67	140			

References:

(1) Dodda, L. S.; Vilseck, J. Z.; Tirado-Rives, J.; Jorgensen, W. L. 1.14*CM1A-LBCC: Localized Bond-Charge Corrected CM1A Charges for Condensed-Phase Simulations. *Journal of Physical Chemistry B* **2017**, *121* (15), 3864–3870. https://doi.org/10.1021/acs.jpcb.7b00272.

(2) Rappé, A. K.; Casewit, C. J.; Colwell, K. S.; Goddard III, W. A.; Skiff, W. M. UFF, a Full Periodic Table Force Field for Molecular Mechanics and Molecular Dynamics Simulations. *Journal of American Chemical Society* **2002**, *114* (25), 10024–10035. https://doi.org/10.1021/ja00051a040.

(3) Hansen, C. M. (2007). *Hansen Solubility Parameters: A User's Handbook*. CRC press.

(4) Kennedy, K. G.; Miles, D. T. Electrochemistry of Ferrocene-Modified Monolayer-Protected Gold Nanoclusters at Reduced Temperatures. *Journal of Undergraduate Chemistry Research* **2004,** *4*, 145.

(5) Louisiana State University Macro Server at The Polymer Analysis Laboratory (PAL), https://macro.lsu.edu/howto/solvents/viscosity.htm, reached on 14.04.2024.

(6) Wu, X.; Cui, X.; Wu, W.; Wang, J., Li, Y.; Jiang, Z. Elucidating Ultrafast Molecular Permeation Through Well□Defined 2D Nanochannels of Lamellar Membranes. *Angewandte Chemie International Edition* **2019**, *58* (51), 18524-18529. https://doi.org/10.1002/anie.201912570

(7) Webpage of Dr. Roland Stenutz, https://www.stenutz.eu/chem/solv24.php, reached on 14.04.2024.

(8) Madani, S. H.; Silvestre□Albero, A.; Biggs, M. J.; Rodríguez□Reinoso, F.; Pendleton, P. Immersion Calorimetry: Molecular Packing Effects in Micropores. *ChemPhysChem* **2015**, *16* (18), 3984-3991.

(9) Watarai, H.; Tanak, M.; Suzuki, N. Determination of Partition Coefficients of Halobenzenes in Heptane/Water and 1-Octanol/Water Systems and Comparison with The Scaled Particle Calculation. *Analytical Chemistry* **1982**, *54* (4), 702-705. https://doi.org/10.1021/ac00241a023

(10) Kang, K. M.; Kim, D. W.; Ren, C. E.; Cho, K. M.; Kim, S. J.; Choi, J. H.; Nam, Y. T.; Gogotsi, Y.; Jung, H. T. Selective Molecular Separation on $Ti_3C_2T_x$-Graphene Oxide Membranes during Pressure-Driven Filtration: Comparison with Graphene Oxide and MXenes. *ACS Applied. Material Interfaces* **2017**, *9* (51), 44687–44694. https://doi.org/10.1021/acsami.7b10932.

(11) Li, Z.-K.; Wei, Y.; Gao, X.; Ding, L.; Lu, Z.; Deng, J.; Yang, X.; Caro, J.; Wang, H. Antibiotics Separation with MXene Membranes Based on Regularly Stacked High-Aspect-Ratio Nanosheets. *Angewandte Chemie International Edition* **2020**, *59* (24), 9751–9756. https://doi.org/https://doi.org/10.1002/anie.202002935.

(12) Shao, D. D.; Zhang, Q.; Wang, L.; Wang, Z. Y.; Jing, Y. X.; Cao, X. L.; Zhang, F.; Sun, S. P. Enhancing Interfacial Adhesion of MXene Nanofiltration Membranes via Pillaring Carbon Nanotubes for Pressure and Solvent Stable Molecular Sieving. *Journal of Membrane Science* **2021**, *623*, 119033. https://doi.org/10.1016/J.MEMSCI.2020.119033.

(13) Wang, J.; Chen, P.; Shi, B.; Guo, W.; Jaroniec, M.; Qiao, S.-Z. A Regularly Channeled Lamellar Membrane for Unparalleled Water and Organics Permeation. *Angewandte Chemie International Edition* **2018**, *57* (23), 6814–6818. https://doi.org/https://doi.org/10.1002/anie.201801094.

(14) Wei, S.; Xie, Y.; Xing, Y.; Wang, L.; Ye, H.; Xiong, X.; Wang, S.; Han, K. Two-Dimensional Graphene Oxide/MXene Composite Lamellar Membranes for Efficient Solvent Permeation and Molecular Separation. *Journal of Membrane Science* **2019**, *582*, 414–422. https://doi.org/10.1016/J.MEMSCI.2019.03.085.

(15) Xing, Y.; Akonkwa, G.; Liu, Z.; Ye, H.; Han, K. Crumpled Two-Dimensional $Ti_3C_2T_x$ MXene Lamellar Membranes for Solvent Permeation and Separation. *ACS Applied Nano Materials* **2020**, *3* (2), 1526–1534. https://doi.org/10.1021/acsanm.9b02322.
